# Supplementary material for: Drug repurposing for aging research using model organisms
Source: Aging Cell. 2017 Jun 16;16(5):1006–15. doi: 10.1111/acel.12626 (PMC5595691; doi:10.1111/acel.12626)
Supplement: Supplementary file 7 — Data S1 Zip‐Archive of all report cards. [file ACEL-16-1006-s007.zip › RC_LGA.pdf]

LGA

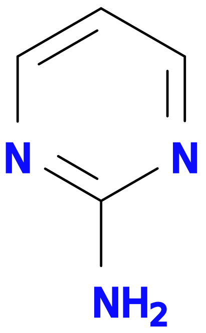

Database identifiers

ChEMBLCompound	CHEMBL88580
CHEBI	38618
ZINC	ZINC00967472
eMolecules	477201

Ranking

	Rank	Score
Drosophila	341/697	0.438
C. elegans	263/591	0.212

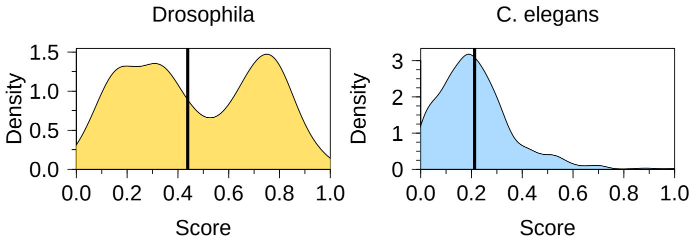

	Ageing implication	Domain conservation	Binding site conservation	Binding affinity	Bioavailability	Lipinski	Promiscuity	Purchasability	Drug approval	**Total**
Drosophila	0.98	0.972	0.847	0.467	(0.9)	0.0	-0.0	0.1	0.0	0.438
C. elegans	0.98	0.967	0.847	0.467	0.301	0.0	-0.0	0.1	0.0	0.212

Names

- **pyrimidin-2-amine**
- 2-aminopyrimidine
- Pyrimidin-2-ylamine

Roles

ChEBI entry 38618 has no roles

Status

Approved drug (according to ChEMBL)	No
Number of Rule of 5 violations	0
Binding affinity to original target in log units (RF-Score prediction)	4.87
Burns *C. elegans* bioavailability prediction	-0.35

Compound Target Characteristics

Heat shock protein HSP 90-alpha

Best gene implication in ageing for this target family came from gene Q18688 annotated in UniProt release 2014_02. Annotation GO 8340 (determination of adult lifespan) was Inferred from Genetic Interaction

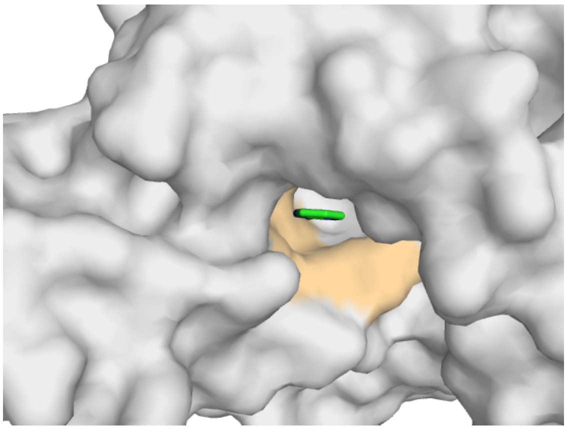

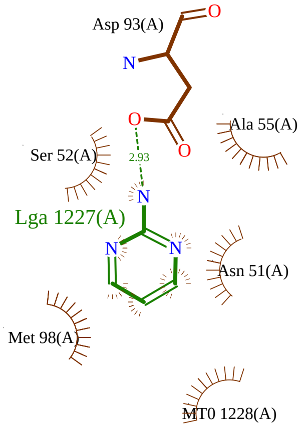

protein	amino acids contacts (binding site)
PDB:2jjc:chainA:P07900	N S A D M T
PDB:2xdu:chainA:P07900	N S A D M T
tr:G3V2J8:G3V2J8_HUMAN	N S A D M T
tr:K9JA46:K9JA46_HUMAN	N S A D M T
sp:P07900:HS90A_HUMAN	N S A D M T
tr:Q6B437:Q6B437_RAT	N S A D M T
sp:P82995:HS90A_RAT	N S A D M T
tr:B7ZC50:B7ZC50_MOUSE	N S A D M T
tr:Q8C2A9:Q8C2A9_MOUSE	N S A D M T
tr:Q3TKB9:Q3TKB9_MOUSE	N S A D M T
tr:Q3TKG0:Q3TKG0_MOUSE	N S A D M T
sp:P07901:HS90A_MOUSE	N S A D M T
tr:Q80Y52:Q80Y52_MOUSE	N S A D M T
sp:P02828:HSP83_DROME	N A A D M T
sp:Q18688:HSP90_CAEEL	N A A D M T
sp:P02829:HSP82_YEAST	N A A D M T

protein	whole protein ident	simil	domain-based ident	simil	contact-based ident	simil
PDB:2jjc:chainA:P07900	1.0	1.0	1.0	1.0	1.0	1.0
PDB:2xdu:chainA:P07900	1.0	1.0	1.0	1.0	1.0	1.0
tr:G3V2J8:G3V2J8_HUMAN	0.24	0.24	0.63	0.63	1.0	1.0
tr:K9JA46:K9JA46_HUMAN	1.0	1.0	1.0	1.0	1.0	1.0
sp:P07900:HS90A_HUMAN	1.0	1.0	1.0	1.0	1.0	1.0
tr:Q6B437:Q6B437_RAT	0.22	0.22	0.85	0.85	1.0	1.0
sp:P82995:HS90A_RAT	0.99	1.0	1.0	1.0	1.0	1.0
tr:B7ZC50:B7ZC50_MOUSE	0.27	0.27	0.86	0.86	1.0	1.0
tr:Q8C2A9:Q8C2A9_MOUSE	0.37	0.37	0.99	1.0	1.0	1.0
tr:Q3TKB9:Q3TKB9_MOUSE	0.75	0.76	0.99	1.0	1.0	1.0
tr:Q3TKG0:Q3TKG0_MOUSE	0.75	0.76	0.99	1.0	1.0	1.0
sp:P07901:HS90A_MOUSE	0.99	1.0	0.99	1.0	1.0	1.0
tr:Q80Y52:Q80Y52_MOUSE	0.99	1.0	0.99	1.0	1.0	1.0
sp:P02828:HSP83_DROME	0.77	0.92	0.85	0.96	0.83	0.85
sp:Q18688:HSP90_CAEEL	0.74	0.89	0.76	0.94	0.83	0.85
sp:P02829:HSP82_YEAST	0.6	0.85	0.68	0.9	0.83	0.85

Hsp83 (FBgn0001233) associated phenotypes

cell cycle defective, cell polarity defective, developmental rate defective, female semi-fertile, lethal - all die before end of pupal stage, lethal - all die during third instar larval stage, partially lethal, partially lethal - majority die, short lived

(Information from FlyBase)

Hsp83 (UniProt:P02828) annotation

Function: Molecular chaperone that promotes the maturation, structural maintenance and proper regulation of specific target proteins involved for instance in cell cycle control and signal transduction. Undergoes a functional cycle that is linked to its ATPase activity. This cycle probably induces conformational changes in the client proteins, thereby causing their activation. Interacts dynamically with various co-chaperones that modulate its substrate recognition, ATPase cycle and chaperone function. Together with Hop and piwi, mediates canalization, also known as developmental robustness, likely via epigenetic silencing of existing genetic variants and suppression of transposon-induced new genetic variation. Required for piRNA biogenesis by facilitating loading of piRNAs into PIWI proteins. (PubMed:21186352, PubMed:22902557).
Subunit: Homodimer (By similarity). Forms a complex with Hop and piwi; probably Hop mediates the interaction between piwi and Hsp83. Interacts with shu. (, PubMed:21186352, PubMed:22902557).
Subcellular location: Cytoplasm.
Induction: In contrast to other major heat shock proteins, this one is also expressed at normal growth temperatures. It is also developmentally expressed during oogenesis.
Domain: The TPR repeat-binding motif mediates interaction with TPR repeat-containing proteins.

(Information from UniProt)

daf-21 (WBGene00000915) associated phenotypes

chemotaxis variant, dauer constitutive, dauer recovery variant, larval lethal, lethal, odorant chemosensory response variant, sluggish, sterile

(Information from WormBase)

daf-21 (UniProt:Q18688) annotation

Function: Molecular chaperone that promotes the maturation, structural maintenance and proper regulation of specific target proteins involved for instance in cell cycle control and signal transduction. Undergoes a functional cycle that is linked to its ATPase activity. This cycle probably induces conformational changes in the client proteins, thereby causing their activation. Interacts dynamically with various co-chaperones that modulate its substrate recognition, ATPase cycle and chaperone function. By stabilizing the receptor-type guanylate cyclase daf-11 or another signal transduction component that regulates cGMP levels, plays a role in dauer forma-

tion and chemotaxis to non-volatile and volatile attractants detected by AWC sensory neurons (PubMed:10790386, PubMed:7828815). Participates in the control of cell cycle progression at the prophase/metaphase transition in oocyte development by ensuring the activity of wee-1.3 kinase, which negatively regulates cdk-1 through its phosphorylation (PubMed:16466390). Regulates yap-1 nuclear export after heat shock treatment (PubMed:23396260). (PubMed:10790386, PubMed:16466390, PubMed:23396260, PubMed:7828815).
Subunit: Homodimer. (UniProtKB:P08238).
Subcellular location: Cytoplasm, perinuclear region (PubMed:12950278). Note=Perinuclear region of somatic cells.
Tissue specificity: In the embryo comma stage, expression is strongly detected in cells of the head region and less so in other areas. In early larvae, expressed in postembryonic germ cells derived from Z2 and Z3 cells and the head region, in both hermaphrodites and males. Under heat stress conditions, larval expression is not only detected in germ cells, but also all over the body. In adult hermaphrodites, expression is localized uniquely in the germ cells. (PubMed:12950278).
Developmental stage: Highly expressed throughout development. (PubMed:10790386).
Domain: The TPR repeat-binding motif mediates interaction with TPR repeat-containing proteins.

(Information from UniProt)
